# Performance Analysis of Inter-Domain Handoff Scheme Based on Virtual Layer in PMIPv6 Networks for IP-Based Internet of Things

**DOI:** 10.1371/journal.pone.0170566

**Published:** 2017-01-27

**Authors:** Chulhee Cho, Jae-Young Choi, Jongpil Jeong, Tai-Myoung Chung

**Affiliations:** 1 College of Information and Communications Engineering, Sungkyunkwan University, Suwon, Kyunggi-do, 440-745, Republic of Korea; 2 Department of Human ICT Convergence, Sungkyunkwan University, Suwon, Kyunggi-do 440-745, Republic of Korea; University of Texas at San Antonio, UNITED STATES

## Abstract

Lately, we see that Internet of things (IoT) is introduced in medical services for global connection among patients, sensors, and all nearby things. The principal purpose of this global connection is to provide context awareness for the purpose of bringing convenience to a patient’s life and more effectively implementing clinical processes. In health care, monitoring of biosignals of a patient has to be continuously performed while the patient moves inside and outside the hospital. Also, to monitor the accurate location and biosignals of the patient, appropriate mobility management is necessary to maintain connection between the patient and the hospital network. In this paper, a binding update scheme on PMIPv6, which reduces signal traffic during location updates by Virtual LMA (VLMA) on the top original Local Mobility Anchor (LMA) Domain, is proposed to reduce the total cost. If a Mobile Node (MN) moves to a Mobile Access Gateway (MAG)-located boundary of an adjacent LMA domain, the MN changes itself into a virtual mode, and this movement will be assumed to be a part of the VLMA domain. In the proposed scheme, MAGs eliminate global binding updates for MNs between LMA domains and significantly reduce the packet loss and latency by eliminating the handoff between LMAs. In conclusion, the performance analysis results show that the proposed scheme improves performance significantly versus PMIPv6 and HMIPv6 in terms of the binding update rate per user and average handoff latency.

## Introduction

Lately, we see that Internet of things (IoT) is introduced in medical services for global connection among patients, sensors, and all nearby things. Healthcare applications utilizing body sensor networks generate a vast amount of data. Cloud computing among with the Internet of Things (IoT) concept is a new trend for efficient managing and processing of sensor data online. Cloud computing is becoming increasingly popular. A large number of data are outsourced to the cloud by data owners [1, 2].

The principal purpose of this global connection is to provide context awareness for the purpose of bringing convenience to a patient’s life and more effectively implementing clinical processes. As IoT is simply M2M (machine to machine) communications, sensor node is reportedly the most appropriate for this new technology. Besides, IPv6 over low power wireless personal area network (6LoWPAN), which can be used in IoT for communicating with sensors, is lately drawing a lot of attention [3]. Hence, the Internet Engineering Task Force (IETF) has set up a working group for IPv6 over Low power Wireless Personal Area Network (6LoWPAN) [4], which is carried out over IEEE 802.15.4 interfaces. In health care, some of the applications that use 6LoWPAN are designed to conduct real-time monitoring of biosignals of a patient being treated at the hospital such as electrocardiogram (ECG), heartbeat, SPo2, blood pressure, weight, and respiratory rate. Importantly, such monitoring has to be continuously performed while the patient moves inside and outside the hospital. Also, to monitor the accurate location and biosignals of the patient, appropriate mobility management is necessary to maintain connection between the patient and the hospital network. Mobility is required to ensure the success of IoT as in health care. To maintain the fault tolerance of the network and ensure free access to their location information while mobile nodes (MNs) move and their locations frequently change, mobility is required [5, 6]. Recently, given the explosive growth of consumer mobile services, efficient use of IP mobility management protocols within a network has been required. Wireless mobile services consume significantly more bandwidth and cost compared with existing services in the current network. The evolution of next-generation IP-based networks, current networks and IP mobility management protocols are linked and integrated; indeed, it seems to be one of most important technologies that enables mobile communication services for the future consumer through IP-based networks [7, 8].

In this paper, we propose and analyze a user-specific, region-specific registration technique for all future IP-based wireless networks to achieve minimal network signal and packet transmission costs to support integrated mobility. When we are given a set of parameters that characterize the operation and operating conditions of Mobile Nodes (MN) for network communication costs, it can be seen that there is an optimal zone that minimizes costs to provide efficient service management operation and mobility of the MN. In this paper, to meet the next generation of wireless/mobile network characteristics mentioned above, PMIPv6 [9] is applied to reduce the signal delay time of the handover cost minimization and Home Agent (HA)/Correspondent Node (CN). In addition, providing an IP-based wireless/mobile network based on the PMIPv6 standard from the structural aspect can provide seamless mobility and minimum arranged overhead. PMIPv6 was intended to reduce the consumption of the core network and provide a form of location privacy by blocking the device connected to the current network. The proposed scheme is based on the virtual layer in the Proxy Mobile IP domain, consisting of a Virtual LMA (VLMA) [10] over a Local Mobility Anchor (LMA) to operate as an HA for the MN. In this paper, the proposed inter-domain handoff scheme to improve the performance of PMIPv6 is based on a virtual layer that uses VLMA. The virtual layer of the proposed scheme is composed of virtual LMA, managed by each of the virtual layers. The proposed scheme allows an MN to move around the MAG boundary of the adjacent LMA domain within VLMA or overlapping areas to which the MN moves. Because handoff does not occur between the LMAs, the proposed scheme greatly reduces the packet loss and delay. In addition, it significantly improves the performance compared with conventional PMIPv6 in terms of the Binding Update (BU) speed per user average handoff delay time.The rest of this paper is organized as follows. In Chapter 2, we describe related research. Chapter 3 describes the proposed scheme based on PMIPv6. Chapter 4 describes the signal cost, packet forwarding and tunneling costs, and modeling and analysis of the total cost of the proposed scheme. Finally, Chapter 5 provides our conclusions.

## Related Work

### Mobile IPv6 for performance improvement

#### Hierarchical Mobile IPv6

**Fig 1:**
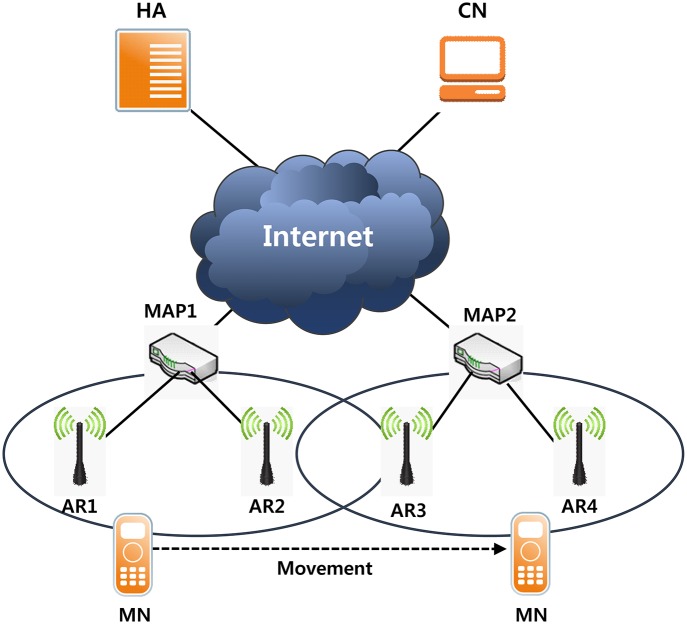
HMIPv6 network architecture.

Hierarchical Mobile IPv6 (HMIPv6) is a protocol for reducing the handover delay of a network Mobile Node (MN) in Mobile IP, as proposed by IETF. HMIPv6 was introduced into a hierarchy in binding management [11] to reduce the signaling load caused by the handover. That is, we define a Mobile Anchor Point (MAP) for locally managing the movement of the MN. When the MN moves from a Mobile IP environment, it transmits a BU, which contains the Care of Address (CoA) to the HA and CN. The CoA in HMIPv6 consists of the CoA of the domain level and the CoA of the link level. The CoA of the domain level is based on the prefix of the MAP domain and is called the Regional Care of Address (RCoA). The CoA of the link level is based on the prefix of the Access Router (AR) and is called the On-Link Care of Address (LCoA). The MN registers the RCoA and LCoA to the MAP and registers the RCoA to its HA and CN. As the MN moves between the AR within an MAP domain, the MN generate only the LCoA and does not generates a new RCoA because the MAP domain has not changed. Thus, movement of the MN in the MAP domain reduces the signaling between the MN and the HA or CN. This is called a Micro Handover when selecting a new MAP-to-MAP domain outside. When the MN is moved to a new MAP domain in HMIPv6, the MN receives a Router Advertisement (RA) message from the new AR [12]. The MN generates aLCoA based on a prefix of the AR, and MN performs Duplicate Address Detection (DAD) for the LCoA itself. The LCoA is newly generated each time the MN moves the AR. The MN generates a new RCoA based on the prefix of the MAP included in the MAP Option of the RA messages. The RCoA address is not changed until the MN has moved to another MAP domain. The MN sends a Local Binding Update (LBU) message to the MAP, including the two addresses after creating the RCoA and LCoA. MAP performs a DAD check for the RCoA address after receiving the BU message. MAP ensures that the RCoA address of the MN is unique within the domain and stores the address of the MN in both their Binding Caches. The MAP stores both addresses of the MN using the Proxy Neighbor Advertisement (PNA) message. MAP, by tunneling, delivers the packet to LCoA of MN intercepts that reach the RCoA of the MN using the PNA message. MN sends a BU message to register the location to its HA after saving the RCoA and LCoA of MN in the Binding Cache. The BU message includes the RCoA as the HoA and CoA. HA sends a Binding Acknowledgment message to the MN after storing the HoA and RCoA of MN. The Destination Address is the RCoA of the MN. MAP is delivered by tunneling the packets to LCoA of the MN. The MN is able to register the location to the CN after the location registration is completed with HA. Fig 1 shows the HMIPv6 network architecture.

#### Proxy Mobile IPv6

**Fig 2:**
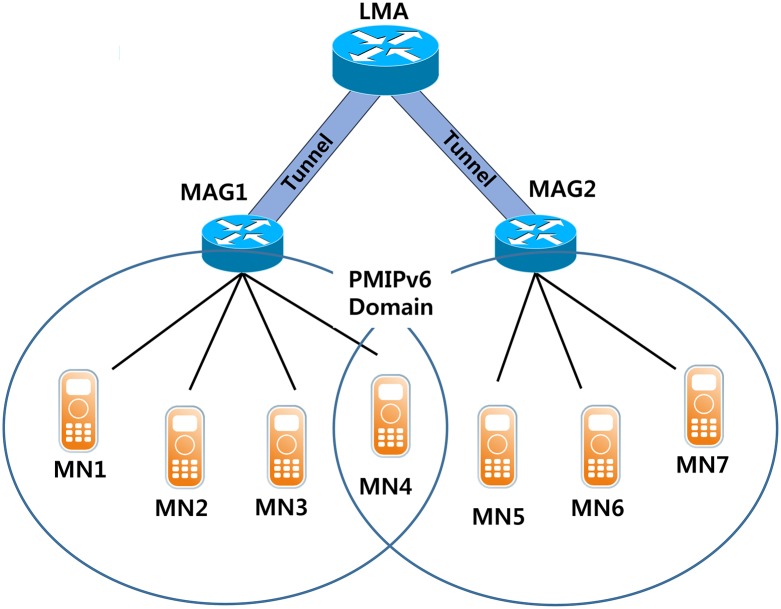
PMIPv6 network architecture.

PMIPv6 is a mobility management scheme developed by IETF for managing the mobility of the MN in the network instead of MN when moving the MN in the Access Network. PMIPv6 is a network-based mobility management protocol that allows MN to change its location without any signal being created. The Mobility Access Gateway (MAG) that supplies the newly introduced mobile services and Local Mobility Anchor (LMA) in PMIPv6 are responsible for managing the movement of the MN in the PMIPv6 domain. MAG is operated in the routers attached to the link and is responsible for processes relating to the movement of the MN. LMA is in operation for a UE to the HA in the Proxy Mobile IP domain [13] to maintain a registration of the MN provided by the MAG, and it manages the routing of data packets to the MN. LMA sends a Proxy Binding Acknowledgment (PBA) message in response to a Proxy Binding Update (PBU) message sent by the MAG assigned to the MN that offers the Home Network Prefix (HNP). Accordingly, when using the Proxy Mobile IP, any modification to the TCP/IP protocol stack of the MN without the MN is able to change the connection without changing the IP address. Fig 2 shows the PMIPv6 network architecture.

**Fig 3:**
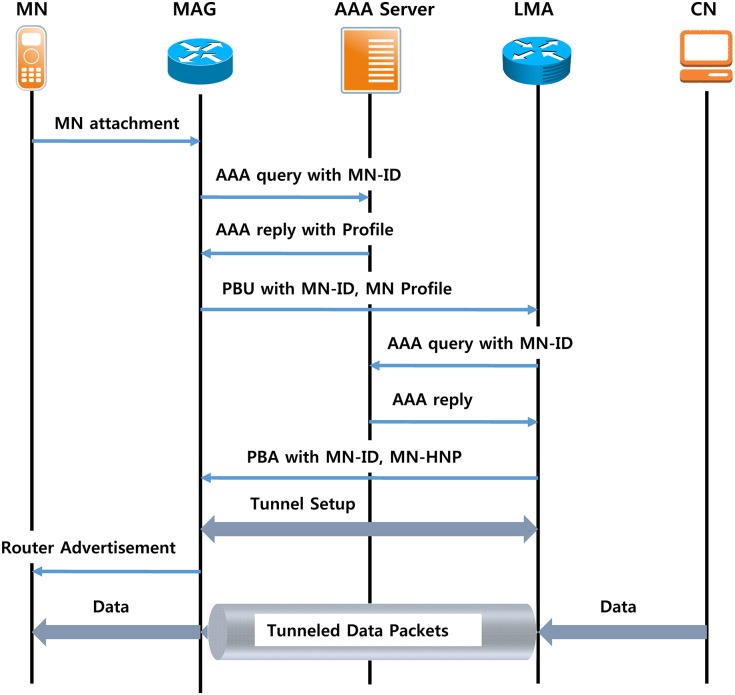
Signaling when a mobile node connects to PMIPv6.

The initial connection procedure for the PMIPv6 domain of the MN is shown in Fig 3. When the MN is connected to the link, MAG, through an MN Attach, acquires the ID and profile information of the MN. The profile information of the MN may be obtained using a Policy Server. Within the profile of MN ID, the IPv6 address of the LMA, and the IP address setting method on the access link are described as essential and may also include the home network address of the further IPv6 MN. When MAG has acquired a profile, it transmits a PBU message to register the current location of the MN to the LMA [14]. LMA receives a PBU and sends a PBA message that contains information on the MN to the MAG HNP to produce a bidirectional IP tunnel between the Binding Cache Entry (BCE) and MAG-LMA to hold the state reached in the MN. If the MAG receives a PBA to set an IP tunnel between MAG-LMA and set the routing table for the data transmission to the MN, thereafter, the MN is an RS/RA procedure on its Home Network Prefix (HNP) after acquiring information, such as the address setting method, to set the IP address.

**Fig 4:**
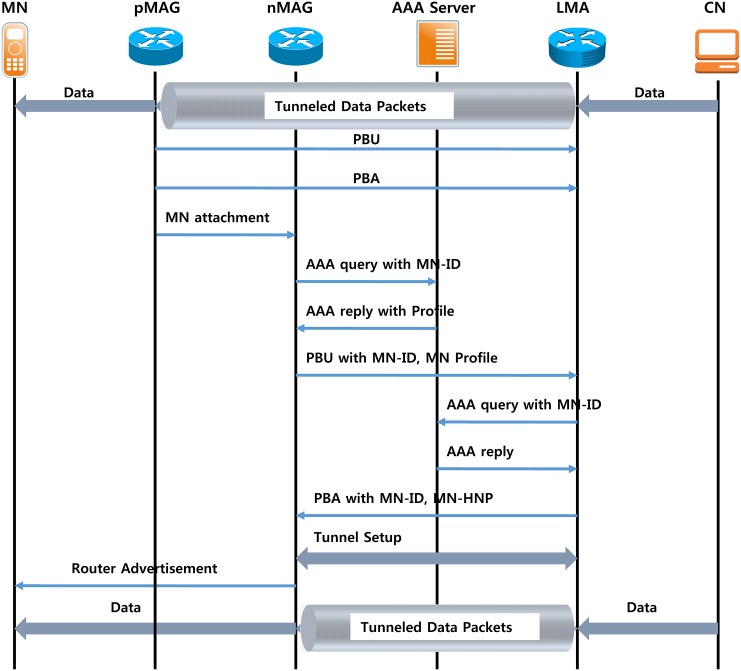
Signaling in a mobile node handoff.

The packets are sent to the MAG via the IP tunnel between MAG-LMA, and the LMA is received and forwarded to the MN after the address set is sent to the MN in the PMIPv6 domain outside the domain. After the transmitting packet from MN is passed to the LMA using an IP tunnel from the MAG, it is transmitted again on the LMA as a destination [15]. The MN in the PMIPv6 domain handoff procedure is shown in Fig 4.

The previous MAG (pMAG) is detected via the MN Detach; the MN is not present on the access link. PBU to the LMA will be notified of the departure of the MN through a message. The LMA performs an operation for deleting the Binding Cache Entry (BCE) related to the MN and sends a PBA. When the MN is connected to the new MAG (nMAG), nMAG performs the initial access procedure of the MN, and the MN sends the HNP information to the MN received during the initial connection via the assigned RS/RA message exchange. Thus, the MN may use a Home of Address (HoA) allocated first. However, packets that are exchanged between the MN and the CN are transmitted via the technique through the PMIPv6 MAG and LMA, as well as the MAG and LMA in the CN of the MN. The transmission scheme through the following alternative routes requires long transfer delays and significant processing costs. Various performance-enhancing techniques have been proposed to address this problem.

### 6LoWPAN

The Internet of Things (IoT) can be implemented by highly constrained devices such as Smart City, Smart Health Care, or Smart Car [5]. These devices have lowperformance properties due to their constraints in terms of memory capacity, computation capability and energy autonomy [16]. For several years, we have seen devices using constrained connectivity and communication capacity in low-power Wireless Personal Area Networks (LoWPANs). To extend the Internet to smart devices, the IETF working group has recently defined IPv6 over low-power Wireless Personal Area Network (6LoWPAN). 6LoWPAN defines an adaptation layer to send IPv6 packets through IEEE 802.15.4 physical and medium access control layers. The 6LoWPAN adaptation layer is needed to reduce the size of IPv6 packets and make it suitable for 127 byte IEEE 802.15.4 frames. 6LoWPAN consists of a header compression scheme, fragmentation scheme and a scheme for framing IPv6 link local addresses [17]. Therefore, the adaptation layer is defined between the IP layer and the MAC layer to transport IPv6 packets over IEEE 802.15.4 links. The adaptation layer is responsible for fragmentation, reassembly, header compression, decompression, mesh routing and addressing for packet delivery under mesh topology [5].

## Proposed Scheme

**Fig 5:**
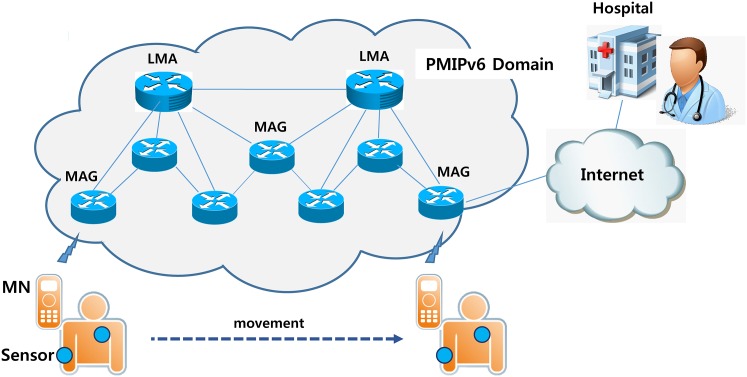
PMIPv6 network architecture for IP-based Internet of Things.

Recently, efficient use of the IP mobility management protocols from the network, given the explosive growth of consumer mobile service, has been required. Wireless mobile services consumes significantly more bandwidth and cost compared with existing services in the current network. IP mobility management is still one of the most difficult research topic despite the development of various protocols by the Internet Engineering Task Force (IETF). Mobile IPv6 (MIPv6) [13] was developed as a host-based mobility management protocols such as for universities and companies and much research was done in the societies. Since then, Fast Mobile IPv6 (FMIPv6) [18] and the Hierarchical Mobile IPv6 (HMIPv6) [19] were developed as extended protocols to complement the weakness of the MIPv6. However, this protocol may not yet have been used effectively because of limitations on the physical network having host-based mobility management protocols that require the mobility processing to the UE. Fig 5 illustrates the PMIPv6 network architecture for the IP-based Internet of Things. To solve the above problem, we propose an inter-domain handoff scheme based on a virtual layer in PMIPv6 networks, which reduces signal traffic during location updates by Virtual LMA (VLMA) on the top original Local Mobility Anchor (LMA) Domain. If the Mobile Node (MN) moves to a Mobile Access Gateway (MAG)-located boundary of an adjacent LMA domain, it changes itself into virtual mode, and this movement will be assumed to be a part of the VLMA domain. In the proposed scheme, MAGs eliminate global binding updates for MN between LMA domains and significantly reduce the packet loss and latency by eliminating the handoff between LMAs.

### Handoff scheme using virtual layer between the LMAs

#### Layer concept

**Fig 6:**
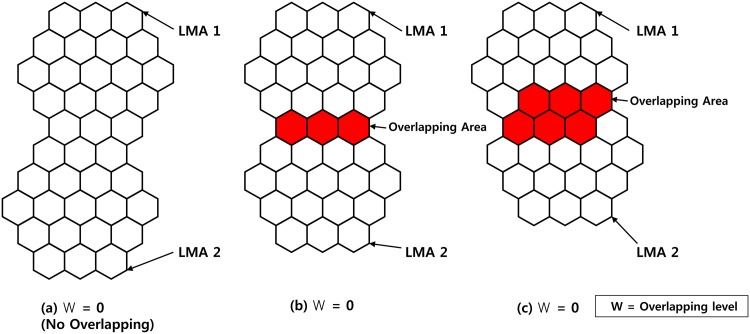
Overlap Function.

The concept of micro-cells was adopted to improve the system capacity in a place with a high user density. However, the smaller cell size is to increase the BU frequency of the MN, and the signaling traffic associated with mobility management increases as a result. Each domain consists of a group of cells, and each cell of the LMA service area belongs to exactly one domain LMA. The location of the MN in a mobile network is confirmed by the LMA in that it belongs to the domain and the MAG to which the MN belongs in the LMA domain, as informed periodically. Additionally, it detects the change in the MN from the LMA domain and sends a BU message to the network. However, the overlap scheme is to prevent the short-term changes due to the MN moving along a boundary of two LMA domains where BU traffic increases. That is, LMA overlapped domain traffic may be reduced (Fig 6). The *w* value is actually the number of lines of overlapping cells and represents the degree of overlap in Fig 6. In non-overlapping Fig 6(a), whenever one of the MN exceeds the LMA domain boundary, there is a need to update the location. If a closed LMA domain overlaps, as in Fig 6(b) to generate an overlap area over the MN, there is only BU. That is, to generate a BU means one of the MNs should exceed the boundary of a cell completely. As shown in Fig 6(c), the MN should overcome a number of cells larger than the size of the overlapped area to send the BU message (in this case, two). This scheme significantly reduces the traffic signal compared with a scheme that does not overlap, but it has the disadvantage that it does not overlap the cells of the LMA domain uniformly. As a result, it is more complex to manage the BU message to the LMA because the overlapped domain, otherwise VLMA, is required for a larger number of schemes. In addition, all BU requests in PMIPv6 are served by the LMA. Thus, the link of the traffic of the LMA should be minimized because it leads to a worse result. In this paper, to reduce the BU ratio effectively and eliminate the overhead, we propose a method of distributing the signal traffic from the LMA to the MAG.

**Fig 7:**
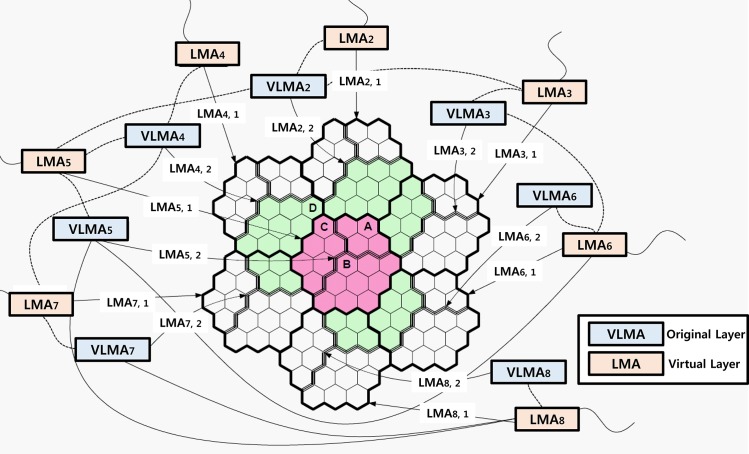
Proposed concept of virtual interface.

This scheme is for the MAG in the boundary zone of the LMA. It is configured to receive the LMA option from all adjacent LMAs and perform the change, not the handoff between the LMA, when the MN is connected with the MAG of the border areas of overlap with the LMA domain. The LMA changes because the MN disconnects after it is moved to another LMA while maintaining the connection to the current LMA. Thus, it is the same procedure as the handoff between LMA; additional packet loss and handoff delay will not occur. If the MN moves to the LMA domain boundary of the adjacent MAG, it changes to virtual mode, and the motion is considered to be within the VLMA domain. Because the MN performs the LMA change within the MAG boundary region, packet loss and additional handoff delay are not generated. The location management scheme proposed in this paper introduces the concept of a virtual layer. As shown in Fig 7, the entire region is divided into seven LMAs, drawn with a thick line (LMA2-LMA8), as mentioned previously, each LMA domain has a single associated VLMA. The original hierarchy of the LMA domain name is a Layer-1, extended VLMA that may overlap. It is important that there is a virtual layer of another part of the region using the three lines named ‘Layer-2’. The size of the VLMA is the same as that of the LMA, and each VLMA is placed in the center of three LMAs and combined. Groups that are managed by the LMA domain are composed of three VLMA domains of Layer-2 and one LMA domain of Layer-1 (Fig 7); LMA_i, j represents LMA i of Layer j.

For example, LMA_5,1 consists of LMA_2,2, LMA_4,2, and part of LMA_5,2. The LMA domain of Layer-1 is connected to three VLMA domains, representing the LMA of Layer-2. LMA_5 is connected with VLMA_2, 4, and 5. The proposed structure is effective to prevent the occurrence of the ping-pong effect if the MN is moved along the boundary of two adjacent LMA domains or the location updates signal traffic through the cell using a virtual layer dispersion.

#### Operating Procedure

Each MN monitors the broadcast message of the MAG. If the current LMA domain is different from the previously registered LMA domain, MN starts the location update to inform the system of the new LMA domain. When incoming session requests from the MN arrive, the system starts responding to register it. The proposed scheme can be implemented by a unique ID assigned to each LMA domain of Layer-1 and -2, covering the service area by homologous LMA domains. Although the original LMA domain partially overlaps with the virtual layer domain of VLMA, each cell is managed by the other two, but the MN is registered in the domain of only one LMA domain. The initial selection is determined depending on the distance to the center of the cell from the cells residing in the two LMA domains. A shorter distance governs the LMA domain selection, whereas it is selected at random when the distance is the same. The MN of cell A in Fig 7 is in both LMA_5,1 and LMA_2,2, but it is registered in LMA_2,2, closer to the center cell. Similarly, the MN is registered in the cell B LMA_5,1. BU is generated when MN is outside the current domain of the LMA. In addition, MN, to prevent the ping-pong effect, is caused to move along the boundary of two adjacent LMA domains and always be registered with the LMA domain different from the previous layer in the extension group. The MN (Layer-1 belongs to the registered LMA_5,1) is in cell B in Fig 7. It is assumed that through cell C (LMA_4,1 and belongs to LMA_4,2), it moves to cell D. Therefore, MN is registered in LMA_4,2, belonging to the Layer-2 non-LMA_4,1. This approach prevents a continuous BU when the MN moves around the boundary cells. BU is required if MN travels through cells C and D each time, but BU is not required if the registered LMA_4,2 belongs to cells C and D. As a result, the overlap scheme reduces traffic signals by the BU. Three LMAs are connected to VLMA: e.g., VLMA_4 in Fig 7 is connected with LMA_4, 5, and 7. The LMA and VLMA handle the traffic of each LMA domain of Layer-1 and -2. BU is not generated when the MN is moving in the three adjacent LMA domains to be processed by the VLMA. Thus, the proposed scheme significantly reduces the traffic going to the LMA in VLMA (performance evaluation results are presented in Chapter 4).

#### Method for selecting LMA by MAG

**Fig 8:**
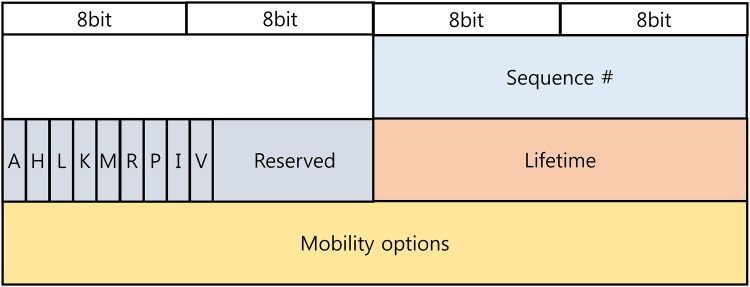
Proxy binding update message.

Fig 8 shows the PBU. The new flag *P* is included in the BU message to register a PBU message in the LMA (Fig 8). The *P* flag is set to 1 for proxy registration and is sent to the MN and set to 0 for registering directly. The variable-length field indicates a length of an integer multiple of 8 octets long, such as the full mobility header length. This field contains zero or more TLV (type, length, and value)-encoding mobility options. Other types of defined options for encoding are described in 6.2 of [13], and the added *R* and *M* flags aregiven in [19] and [20], respectively.

In this proposed scheme, modifying some of the Handoff Indicator options in the message comprises new extension flags *I* and *V*, and the extended flag has the following properties. The *I* flag indicates the classification of the layer (0 indicates the first layer and means a virtual layer), and the *V* flag indicates that the MAG signaled a physical connection with VLMA. The MAG is in this case will be considered in the Layer-1 LMA and will be ignored for any option that LMA cannot understand. Network operators may place several LMA at different distances from the MAG within the respective domain. The LMA domain is used to select PMIPv6 in Fig 4, as in the initial network entry process of the MN, so that MAG has the Policy Profile of the MN. MN locates the MAG by the profile information of the MN (MN’s ID, provided MAG, LMA network address, before and after the move circumstances of the MN), the process proceeds to the search process to select the optimal LMA between the two layers of the proposed method. After the search process of MAG sends a PBU message to the LMA to finish the binding, it uses the new expansion flag in the PBU message, described above. Set the Proxy Care of Address (pCoA) of the layer corresponding to the LMA to receive the information required for basic PMIPv6 operation. That is, the search process selects for MAG an appropriate LMA in the profile of the MN. The received information consists of the preference values of LMA, a valid lifetime field, the distance field, IP address fields, and some flags: the I flag, which distinguishes from the virtual layer and the hierarchy, and the *V* flag that determines the VLMA and physical connections. MAG will send a return value passed because the search process, again with LMA and preference values corresponding to the MAG position information of the PBU message in PMIPv6, is registered with the PBU message. The search procedure is described in detail as follows. Depending on the position of the MAG where the MN approaches the boundary, the value of preference is also reduced by one and transmitted to the MN through an RA message.

**Fig 9:**
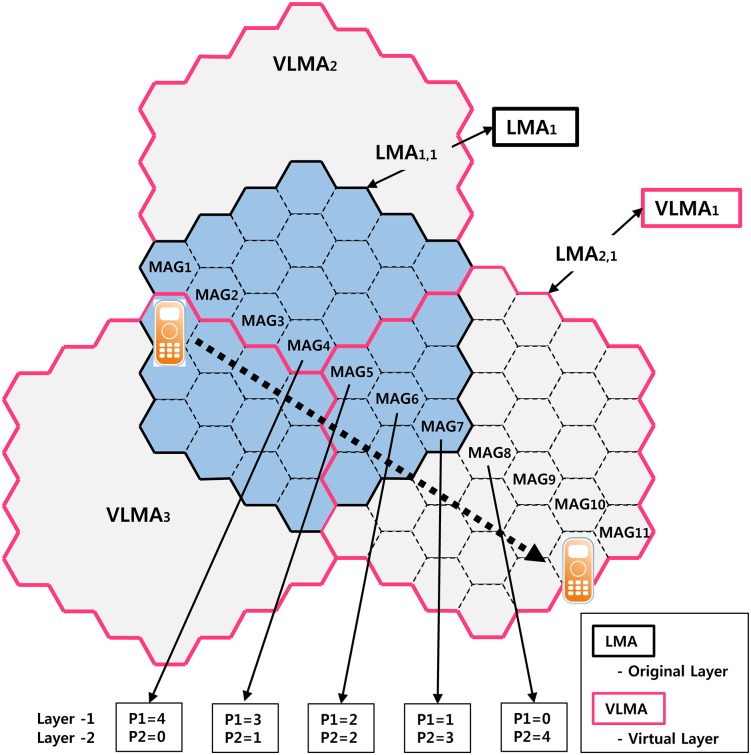
Preference change according to movement of the mobile node.

**Fig 10:**
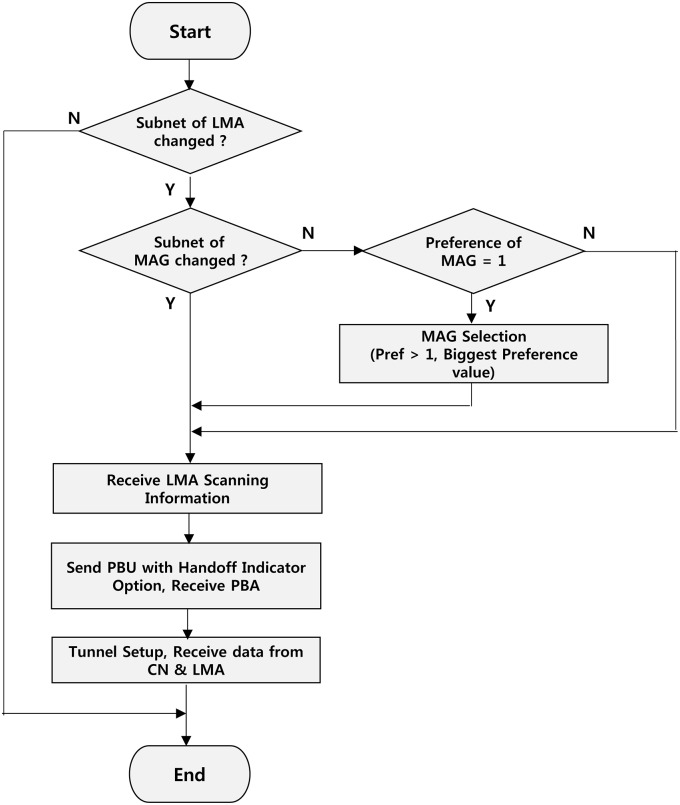
Operation flowchart according to the movement of a mobile node.

In the proposed scheme, when MAG is located on the border, the value of preference is always maintained at 1 when the MN is connected to the network; the MAG is aware that the MN has arrived at the boundary of the LMA domain. MAG causes MN to check the physical connection (*V* flag) between LMA1 and VLMA1 and then execute the LMA change. As shown in Fig 9, the value of LMA1 preference is 1, and the value of VLMA1 preference is 3 in the MAG7-belonging MN. Therefore, the value of the *I* flag is 0 from LMA1 and from VLMA1 is 1, so VLMA1 is selected. After the current link to VLMA1 is changed, the physical connection is established, and then the *V* flag values that are physically connected to the current LMA domain are checked. If there is a physical connection, perform the LMA change. If two LMA domains are overlapping and MN is in the area, such as an overlapping region of MAG5, MAG6, and MAG7, MN belongs to the MAG and will receive transmission information via the search process using the MN Policy Profile from LMA1 and VLMA1. Preference values are used to hide the location information of MAG of the LMA domain; MAGs are sent to the LMA through the PBU message after Preference is reduced to 1. For example, when the MN is in MAG7, the preference values are 1 through the search process from LMA1 of Layer-1; after VLMA1 of Layer-2 receives the preference value of 3 through the procedure, a PBU updates to LMA1 and VLMA1. When the MN moves from MAG6 to MAG7, handoff occurs between LMAs and packets are received from the CN via LMA1 and MAG7 (passage 1). MAG7 is located on the border MAG in the overlap region from the MN changes to VLMA1 from LMA1. The data packets pass through MAG7 and VLMA1 (passage 2) and are transmitted to the MN. MAG7 is located at the domain inside, so MAG7 does not incur handoff between LMAs. Fig 10 shows an operational flowchart of the LMA according to the movement of the MN by the proposed scheme. Some of the LMAs receive navigation information when the subnet of the MAG is changed. It selects the LMA and inserts navigation information to the Handoff Indicator option. After transmitting the PBU and after receiving a PBA, sequentially, it receives data from the CN and LMA via the tunnel.

**Fig 11:**
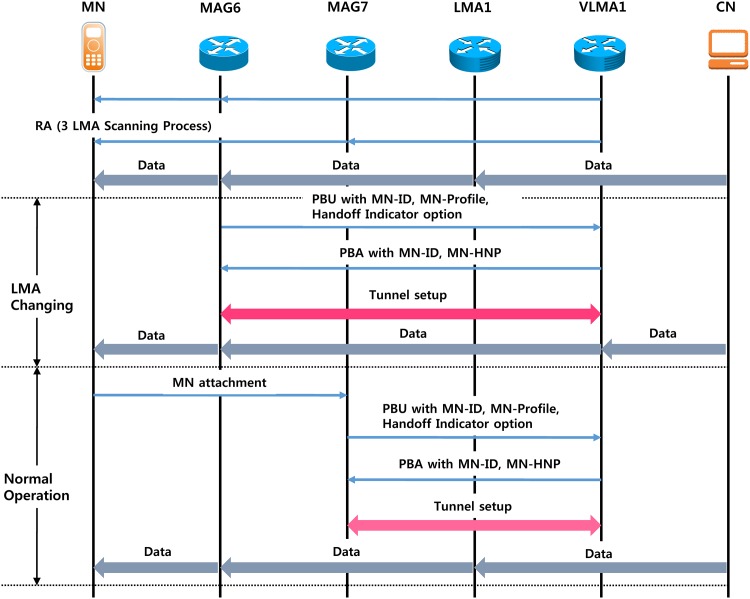
Message flow.

Alternatively, to change the subnet of the LMA instead of the subnet of MAG owing to the movement of the MN, MAG sends a PBU to the insert to change the navigation information, and the LMA receives the data through the tunnel after receiving a PBA. Additionally, when the MAG serving the MN does not change the subnet of the LMA, it checks the preference value. If the value is 1, it changes the LMA, and the selected LMA domain has a value greater than 1. Messages flow to improve handoff performance by changing the LMA (Fig 11), and the MN moves to MAG7 in MAG6 based on the network connection mode of Fig 9. MAG6 and MAG7 will receive LMA1 and VLMA1 from the search. When the MN is in MAG7, the value of the LMA1 preference is 1, and the value of the VLMA1 preference is 3. When the MN of LMA1 is changing the MAG, the MAG keeps the MN using the LMA navigation information to select the new LMA.

**Fig 12:**
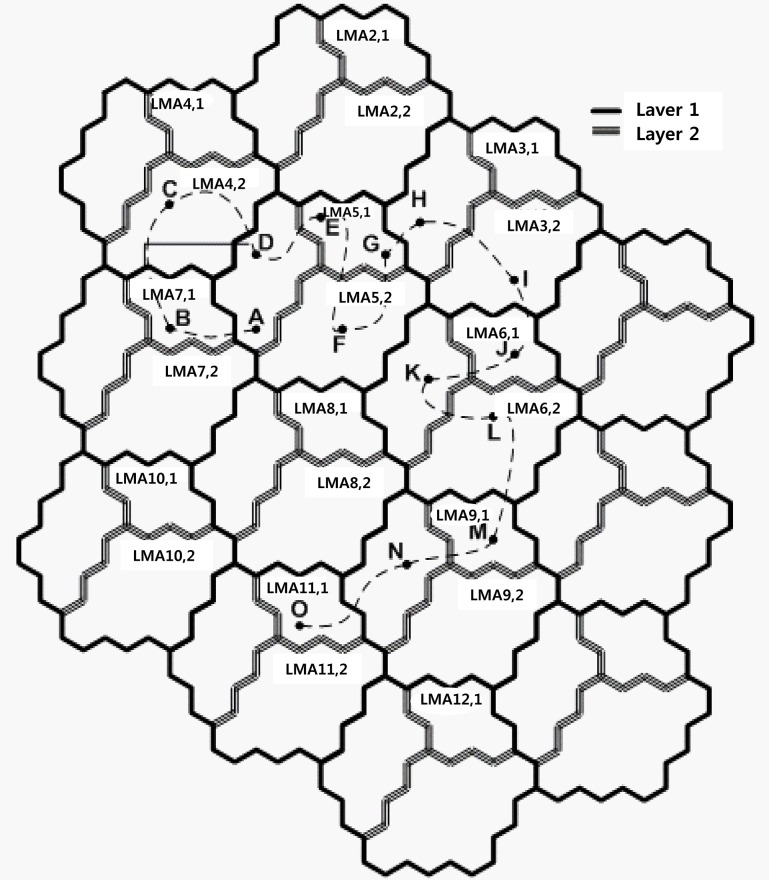
Case of the movement of a mobile node.

The MAG registers the preference values, and corresponding values are transmitted in the search process after a PBU message to the location information of the MAG with the PBU message changes to the LMA selected. Additionally, because the MN, when the MN receives the RA, has already changed from LMA1 to VLMA1, MN completes the handoff, and MAG sends a PBU message only to VLMA1. When the MN is in MAG6 or MAG7 and LMA1 and VLMA1 to both, the MN performs a search to select the current serving MAG, and LMA receives the respective information. Fig 12 shows the change in LMA according to the movement of the MN. The first MN is located in A and binds with LMA_5.1. When the MN moves to B, it is changed to the time LMA_4.2. When the MN moves to D via C at position B and is in LMA_4.2, LMA to the BU is not required. If MN arrives in E, there is a need to change LMA and change the LMA in VLMA. MN is required to update the location while moving to G. Table 1 describes the location update executed as the MN moves to O from A. Thus, the proposed scheme performed LMA changes 9 times, and BU must perform the changes from MAG to LMA 3 times. If BU techniques based on a virtual layer are not used, BU must perform the changes from MAG to LMA 7 times.

**Table 1:** Handoff difference between PMIPv6 and the proposed scheme when MN is moving.

	Movement	Registered LMA	Proposed Scheme	PMIPv6
1	*A* → *B*	LMA_4,2	LMA Changing	LMA handover
2	*B* → *C*	LMA_4,2	None	LMA handover
3	*C* → *D*	LMA_4,2	None	LMA handover
4	*D* → *E*	LMA_5,1	LMA Changing	None
5	*E* → *F*	LMA_5,1	None	None
6	*F* → *G*	LMA_5,1	None	None
7	*G* → *H*	LMA_2,2	LMA Changing	LMA handover
8	*H* → *I*	LMA_3,1	LMA Changing, LMA handover	None
9	*I* → *J*	LMA_3,2	LMA Changing	LMA handover
10	*J* → *K*	LMA_6,1	LMA Changing	None
11	*K* → *L*	LMA_6,1	None	None
12	*L* → *M*	LMA_6,2	LMA Changing, LMA handover	LMA handover
13	*M* → *N*	LMA_9,1	LMA Changing	None
14	*N* → *O*	LMA_8,2	LMA Changing	LMA handover

## Performance Evaluation

### Network model with mobility message

#### Network Model for Performance Evaluation

**Fig 13:**
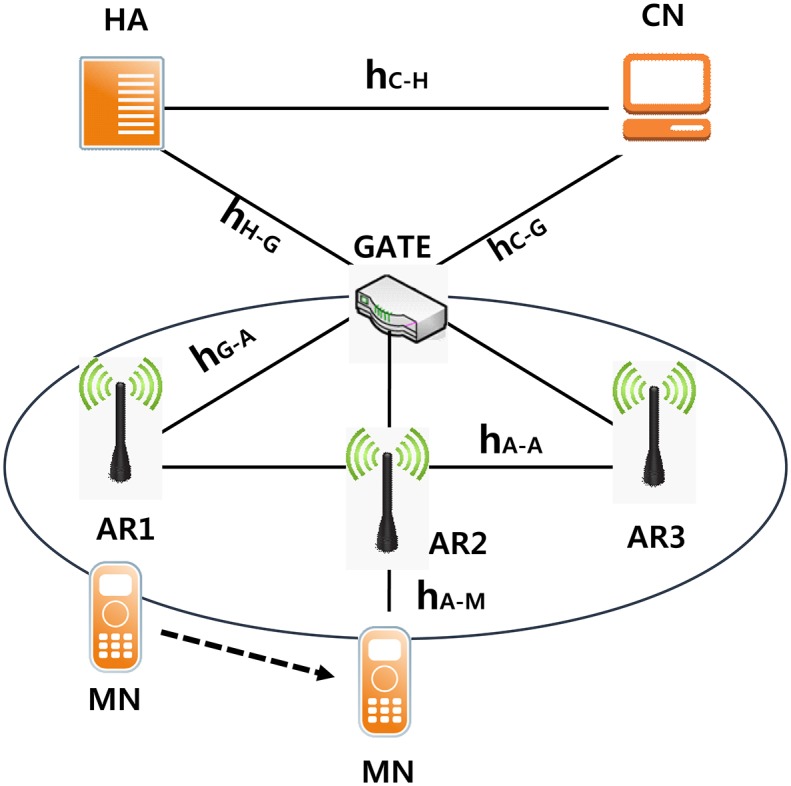
Case of the movement of a mobile node.

Fig 13 shows a network that was considered in the cost model through comparison with other schemes (HMIPv6, PMIPv6) to evaluate the performance of the scheme proposed in Chapter 3. GATE is assumed to be LMA in MAP and PMIPv6 in HMIPv6. It is assumed that MAG and AR are the same. The following are definitions of the paths shown in Fig 13.

- *h*_*c*−*H*_: The average value of the number of hops between the CN and HA
- *h*_*c*−*G*_: The average value of the number of hops between the CN and the gate
- *h*_*H*−*G*_: The average number of hops between the HA and the gate
- *h*_*G*−*A*_: The average value of the number of hops between the gate and the AR
- *h*_*A*−*A*_: The average value of the number of hops between neighboring AR
- *h*_*A*−*M*_: The average number of hops between the AR and MN

#### Mobility Messages

The byte sizes of the messages used in HMIPv6, PMIPv6 [21, 22], and the proposed scheme are as follows.

- *L*_*BU*−*HA*_: BU message sent by the HA in size MN, 56.
- *L*_*BAck*−*HA*_: BAck message size, 56.
- *L*_*BU*−*CN*_: BU message size sent to the CN in MN, 66.
- *L*_*LBU*−*MAP*_: LBU message size sent to the MAP in MN, 56.
- *L*_*LBAck*−*MAP*_: LBAck message size, 56.
- *L*_*PBU*−*LMA*_: PBU message sent to LMA size in MAG, 76.
- *L*_*PBAck*−*LMA*_: PBAck message size, 76.
- *L*_*HoTI*_: Home test initialization (HoTI) message size, 64.
- *L*_*CoTI*_: Co-test initialization (CoTI) message size, 64.
- *L*_*HoT*_: Home Test (HoT) message size, 74.
- *L*_*CoT*_: Co-test (CoT) message size, 74.
- *L*_*RtSol *Pr_: Router solicitation for proxy advertisement (RtSolPr) message size, 52.
- *L*_Pr* RtAdv*_: The proxy router advertisement message size, 80.
- *L*_*HI*_: The handover initiation (HI) message size, 52.
- *L*_*HAck*_: Handover confirmation (HAck) message size, 52.

A cost analysis model was developed for evaluating the performance of the proposed IP mobility management protocol. Signal cost $C_{BU}^{\left(\cdot \right)}$ is the mobility signal overhead accumulated to support the mobility service of the MN. That is, $C_{BU}^{\left(\cdot \right)}$ is calculated as the product of the hop size of the distance and mobility signaling messages [22, 23]. $C_{PD}^{\left(\cdot \right)}$ is the cumulative cost of packet delivery traffic overhead caused by the transmission of a packet in the routing path. That is, $C_{PD}^{\left(\cdot \right)}$ is calculated as the product of the data packet size and the hop distance. $C_{PT}^{\left(\cdot \right)}$ is similar to the $C_{PD}^{\left(\cdot \right)}$ packet tunneling cost but is used mainly to examine the tunneling overhead. $C_{PT}^{\left(\cdot \right)}$ is calculated as the product of the IPv6 tunneling distance and hop size. Finally, the total cost of $C_{T}^{\left(\cdot \right)}$ is defined as the sum of $C_{BU}^{\left(\cdot \right)}$ and $C_{BU}^{\left(\cdot \right)}$ and $C_{PD}^{\left(\cdot \right)}$ [24].

#### HMIPv6

One of the MNs in HMIPv6 is managed precisely by the MAP. Because MN is used to set up a local CoA in the new access network of a given MAP domain, MN is updated only if there is new location information for MAP. This is because the MAP functions as a local HA for the MN, so it registers the movement of the MN to the HA and the CN for a given transmission MAP domain. Movement of the MN in the HMIPv6 access network is managed by the MAP, which is limited from the inside domain handover. Thus, signal charges for HMIPv6 $C_{BU}^{\left(HMIPv6\right)}$ are equal to Eq (1).

$$\begin{array}{ccc} C_{BU}^{\left(HMIPv6\right)} & = & L_{LBU-MAP}\left(\alpha h_{G-A}+\beta h_{A-M}\right)\\ & & +L_{LBAck-MAP}\left(\alpha h_{G-A}+\beta h_{A-M}\right) \end{array}$$ (1)

where *α* and *β* are weighting factors for a wired link and a wireless link, respectively. They are used to emphasize the link stability. Packet delivery cost for $C_{PD}^{\left(HMIPv6\right)}$ in HMIPv6: the data packets are sent from the CN to the MN, whereas the MN arrives smoothly, staying in the MAP domain. That is, data packets for the MN are always sent on a route directly by bypassing the HA. However, in HMIPv6, MAP is tunneled to the HA and the CN for the transmission of data packets for the MN. That is, when the data packet is sent from the CN to the MN that arrives in the MAP, MAP compresses the data packets sent to the MN. Therefore, $C_{PD}^{\left(HMIPv6\right)}$ is the same as Eq (2).

$$\begin{array}{ccc} C_{PD}^{\left(HMIPv6\right)} & = & \lambda_{S}E\left(S\right)P_{D}^{HMIPv6} \end{array}$$ (2)

where *λ*_*S*_ is the average session arrival rate at the MN’s wireless interface, *E*(*S*) is the average session length in packets, $P_{D}^{\left(HMIPv6\right)}$ is the path directly in HMIPv6 and is the same as in Eq (3).

$$\begin{array}{c} P_{D}^{\left(HMIPv6\right)}=\alpha h_{C-G}+\varpi \alpha h_{G-A}+\varpi \beta h_{A-M} \end{array}$$ (3)

where ϖ is the IPv6 tunneling overhead. The packet tunneling cost for HMIPv6 $C_{PT}^{\left(HMIPv6\right)}$ is equal to Eq (4).

$$\begin{array}{c} C_{PT}^{\left(HMIPv6\right)}=\lambda_{S}E\left(S\right)PT_{D}^{\left(HMIPv6\right)} \end{array}$$ (4)

where $PT_{D}^{\left(HMIPv6\right)}$ is the path directly in HMIPv6 as the tunneling overhead and is given by Eq (5).

$$\begin{array}{c} PT_{D}^{\left(HMIPv6\right)}=\varpi \alpha h_{G-A}+\varpi \beta h_{A-M} \end{array}$$ (5)

The total cost for HMIPv6 $C_{T}^{\left(HMIPv6\right)}$ is equal to Eq (6).

$$\begin{array}{c} C_{T}^{\left(HMIPv6\right)}=C_{BU}^{\left(HMIPv6\right)}+C_{PD}^{\left(HMIPv6\right)} \end{array}$$ (6)

#### PMIPv6

As with HMIPv6, PMIPv6 precisely manages the movement of the MN. However, unlike HMIPv6, which allows the connection point of the entity to be changed, the general MN PMIPv6 domain provides mobility services, such as LMA and MAG. Some mobility signaling messages sent from the MN do not exist in PMIPv6. Considering this, the signal is equal to the cost of $C_{BU}^{\left(PMIPv6\right)}$ for the PMIPv6 Formula (7).

$$\begin{array}{ccc} C_{BU}^{\left(PMIPv6\right)} & = & 2L_{PBU-LMA}\alpha h_{G-A}+2L_{PBAck-LMA}\alpha h_{G-A} \end{array}$$ (7)

The cost of the signal is reduced compared with the formula for $C_{BU}^{\left(HMIPv6\right)}$ shown in Eq (1). This is when the MAG detects the movement of the MN in the access network, on behalf of the MN, and sends a PBU message. However, as shown in [9], a dual mobility signal is generated. MN sends a registration cancellation PBU message to the LMA, announcing the release of the MN in the access network managed by the previous MAG (pMAG) as the connection point is changed. Of course, the new MAG (nMAG), by sending a PBU message, detects the movement of the MN to register the MN to the LMA. The packet transmission cost in PMIPv6 is shown in Eq (8).

$$\begin{array}{c} C_{PD}^{\left(PMIPv6\right)}=\lambda_{S}E\left(S\right)P_{D}^{\left(PMIPv6\right)} \end{array}$$ (8)

In the formula above, the $P_{D}^{\left(PMIPv6\right)}$ route cost directly from PMIPv6 is the same as in Formula (9).

$$\begin{array}{c} P_{D}^{\left(PMIPv6\right)}=\alpha h_{C-G}+\varpi \alpha h_{G-A}+\beta h_{A-M} \end{array}$$ (9)

The packet tunneling cost $C_{PT}^{\left(PMIPv6\right)}$ for PMIPv6 is shown in Eq (10).

$$\begin{array}{c} C_{PD}^{\left(PMIPv6\right)}=\lambda_{S}E\left(S\right)PT_{D}^{\left(PMIPv6\right)} \end{array}$$ (10)

where $PT_{D}^{\left(PMIPv6\right)}$ is the path directly in the PMIPv6 tunnel overhead, as in Formula (11).

$$\begin{array}{c} PT_{D}^{\left(PMIPv6\right)}=\varpi \alpha h_{G-A} \end{array}$$ (11)

The total cost for the PMIPv6 $C_{T}^{\left(PMIPv6\right)}$ is equal to Eq (12).

$$\begin{array}{c} C_{T}^{\left(PMIPv6\right)}=C_{BU}^{\left(PMIPv6\right)}+C_{PD}^{\left(PMIPv6\right)} \end{array}$$ (12)

#### Proposed Scheme

Our proposed method is to minimize the occurrence of the MN handoff when moving by adding a virtual layer on PMIPv6. That is, in the case of the PMIPv6 techniques, when the handoff occurs when the MAG serves the MN to connect through the VLMA Preference according to the value of the MAG sends a PBU to the LMA change procedure changes the previous LMA, the LMA receives an ACK from the handoff receiving a PBU to VLMA already known, without the need to complete and is transmitted to the PBA. Thus, the signal cost $C_{BU}^{\left(PMIPv6\right)}$ is equal to Formula (13).

$$\begin{array}{ccc} C_{BU}^{\left(PMIPv6\right)} & = & L_{PBU-LMA}\alpha h_{G-A}+L_{PBAck-LMA}\alpha h_{G-A} \end{array}$$ (13)

The signal cost from the above equation is reduced, compared with $C_{BU}^{\left(PMIPv6\right)}$ as shown in Eq (7). This is because sending a PBU message affects only nMAG. A dual-mobility signal is not generated as in PMIPv6. There is no need to transmit a registration cancellation PBU message to the LMA for the MN because it does not change the connection point of the separation process procedure of the MN in the access network managed by the pMAG. NMAG also registers the MN, sending a PBU message by detecting the movement of the MN to the LMA. As with PMIPv6, the data packets sent from the CN to the MN are transferred smoothly to the MN. MAG tunnels to the LMA after receiving the first data packet. The ends of the bidirectional tunnel established between LMA and MAG are called the LMA address, as a secondary proxy address, and the MAG address, respectively, and $C_{PD}^{\left(PMIPv6\right)}=\lambda_{S}E\left(S\right)P_{D}^{\left(PMIPv6\right)}$ is the same as in Eq (14).

$$\begin{array}{c} C_{PD}^{\left(PMIPv6\right)}=\lambda_{S}E\left(S\right)P_{D}^{\left(PMIPv6\right)} \end{array}$$ (14)

where $P_{D}^{\left(PMIPv6\right)}$ is directly in the path cost and is equal to the PMIPv6 proposed Formula (15).

$$\begin{array}{c} P_{D}^{\left(PMIPv6\right)}=\alpha h_{C-G}+\varpi \alpha h_{G-A}+\beta h_{A-M} \end{array}$$ (15)

The packet tunneling cost $C_{PT}^{\left(PPMIPv6\right)}$ for the proposed PMIPv6 is shown in Eq (16).

$$\begin{array}{c} C_{PD}^{\left(PPMIPv6\right)}=\lambda_{S}E\left(S\right)PT_{D}^{\left(PPMIPv6\right)} \end{array}$$ (16)

In the formula above, $PT_{D}^{\left(PPMIPv6\right)}$ is the tunneling path overhead directly equal to that of PMIPv6 proposed in Eq (17).

$$\begin{array}{c} PT_{D}^{\left(PPMIPv6\right)}=\varpi \alpha h_{G-A} \end{array}$$ (17)

The total cost of the proposed $C_{T}^{\left(PPMIPv6\right)}$ of PMIPv6 is shown in Eq (18).

$$\begin{array}{c} C_{T}^{\left(PPMIPv6\right)}=C_{BU}^{\left(PPMIPv6\right)}+C_{PD}^{\left(PPMIPv6\right)} \end{array}$$ (18)

### Results of Numerical Analyses

This section shows the cost analysis of the mobility management protocol. Assuming that the coverage of the MAG circle exceeds the boundary of the MN, which is equal to the ratio *μc* Formula (19) [22], then

$$\begin{array}{c} \mu_{C}=\frac{2v}{\pi R} \end{array}$$ (19)

In the formula above, *ν* is the average velocity of the MN, and R refers to the coverage radius of the MAG.

#### Parameter Setting

We use the default value of the variable as follows: The average value of the number of hops between the CN and HA is *h*_*C*−*H*_ = 4. The average value of the number of hops between the CN and the gate is *h*_*C*−*G*_ = 6. The average number of hops between the HA and the gate is *h*_*H*−*G*_ = 4. The average value of the number of hops between the gate and the AR is *h*_*G*−*A*_ = 4. The average number of hops between the AR and MN is *h*_*A*−*M*_ = 1. the average session length in packets is *E*(*S*) = 10. Inter-LMA handoff probability is *α* = 1. Intra-LMA handoff probability is *β* = 1.5. the ratio of data packets going through HA is *ω* = [0.1, 0.9]. the IPv6 tunneling overhead is ϖ=40bytes[6,10].

#### Signal Cost

**Fig 14:**
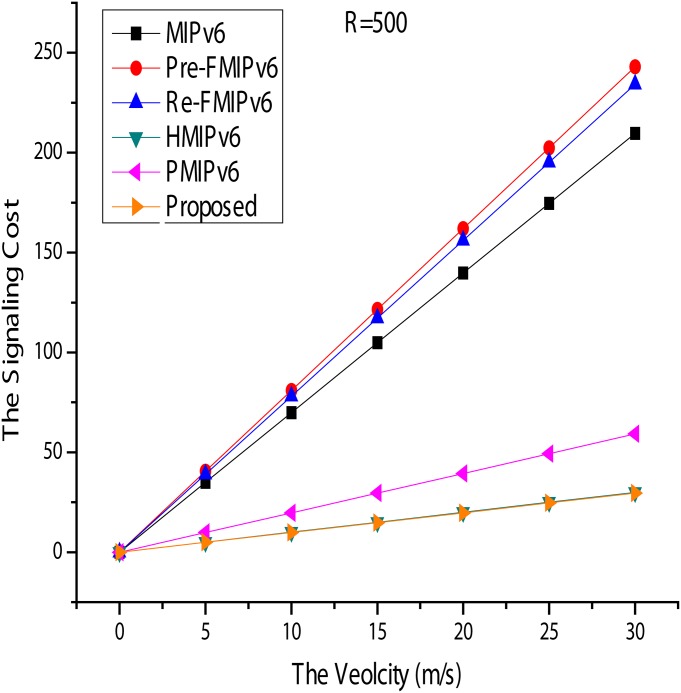
Signal Cost for Velocity.

**Fig 15:**
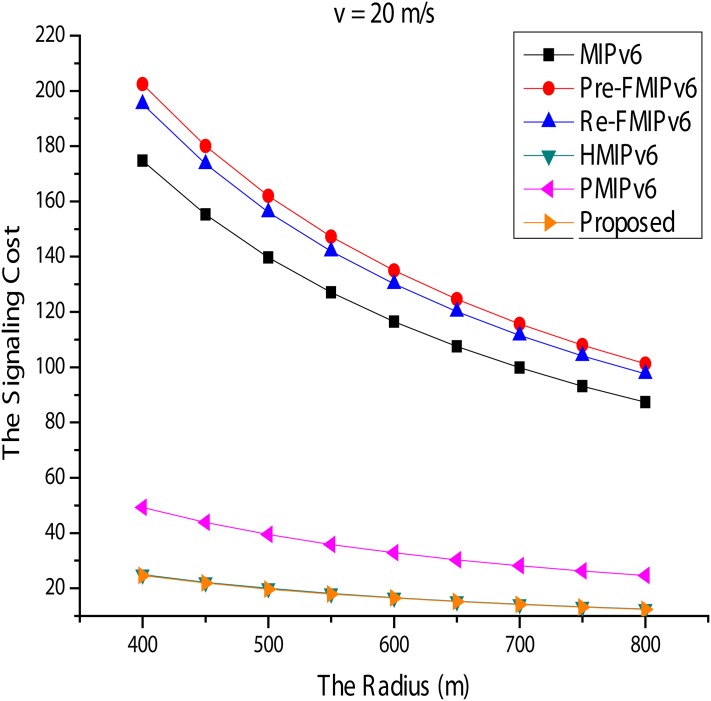
Signal Cost for Radius.

Figs 14 and 15 show the signal cost of the mobility management protocols. R is fixed to 500*m*, and *ν* is varied from 0 to 30*m*/*s*. This protocol provides local mobility services, such as HMIPv6, and PMIPv6 in Fig 15 shows the result of good performance swings. When supporting mobility services, it incurs less mobility signal cost. As *ν* increases, the signal cost also increases. However, when HMIPv6, PMIPv6, and the proposed scheme are compared, the proposed scheme incurs much less signal cost. Mobility services for the MN are managed locally by the MAP in HMIPv6. While the MN changes the connection point in a given HMIPv6 domain, the only exchange of messages is between the MN and the LBU and LBAck MAP.

In addition to the MN in the PMIPv6 mobility services and PBAck, only a PBU message is exchanged between the MAG and the LMA. The proposed scheme shows better performance than PMIPv6. Next, *ν* is fixed at 20*m*/*s*, and R is in the range of 400-800*m*. As shown in Fig 14, HMIPv6, PMIPv6, and the proposed method produce better results than other mobility management protocols. With the MN and MN of FMIPv6 within MIPv6, a BU message is sent to the HA, and the CN must be updated each time the connection point is changed to the new location information. However, HMIPv6, PMIPv6, and the MN of the proposed technique are managed locally, so mobility signals are minimized. Furthermore, if the proposed method and PBAck PBU message exchange improved more than the number of PMIPv6 and showed slightly better performance than HMIPv6. Additionally, for FMIPv6 in Figs 14 and 15, it can be seen that it is the highest consumption protocol. This phenomenon is due to the additional mobility signal being generated during the handover and the buffering mechanism for the rapid handling.

#### Packet delivery cost

**Fig 16:**
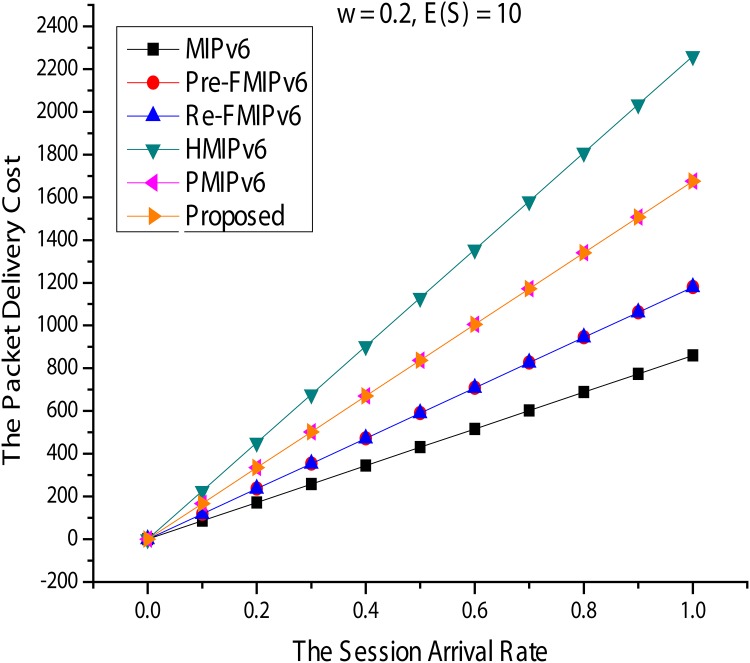
Packet Delivery Cost for Session Arrival Rate.

**Fig 17:**
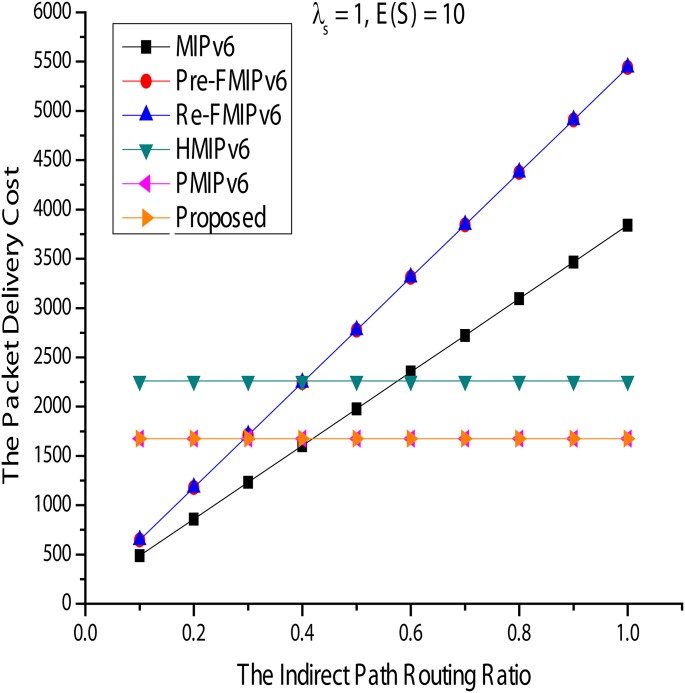
Packet Delivery Cost for Indirect Path Routing Ratio.

Fig 16 shows the packet delivery cost for Fig 17. In Fig 17, *ω* and *E*(*S*) are fixed at 0.2 and 10 and, compared with *λs*, show the variation in packet transmission cost. As *λs* increases, the cost of any mobility management protocol packet transfer is also increased, exponentially. However, in MIPv6, the analysis shows better performance than the others because the packets are sent to the transmission path routed almost directly from CN. Next, *λs* and E(S) are fixed at 1 and 10, respectively, and *ω* is set from 0.1 to 1. In Fig 17, HMIPv6, PMIPv6, and the proposed method do not affect anything in *ω*, whereas MIPv6 and FMIPv6 have a tremendous impact on *ω*. *ω* in MIPv6 and FMIPv6 for packet transmission costs increases dramatically. This is because the quantity of data packets going through the indirect path also increases. Additionally, the packets will be tunneled overhead for FMIPv6 consumption more than the others.

#### Packet tunneling costs

**Fig 18:**
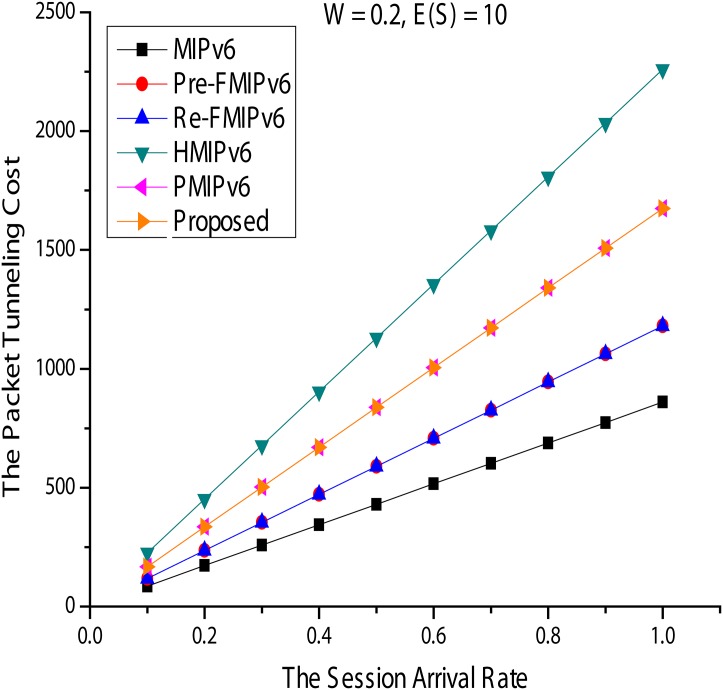
Packet Tunneling Cost for Session Arrival Rate.

**Fig 19:**
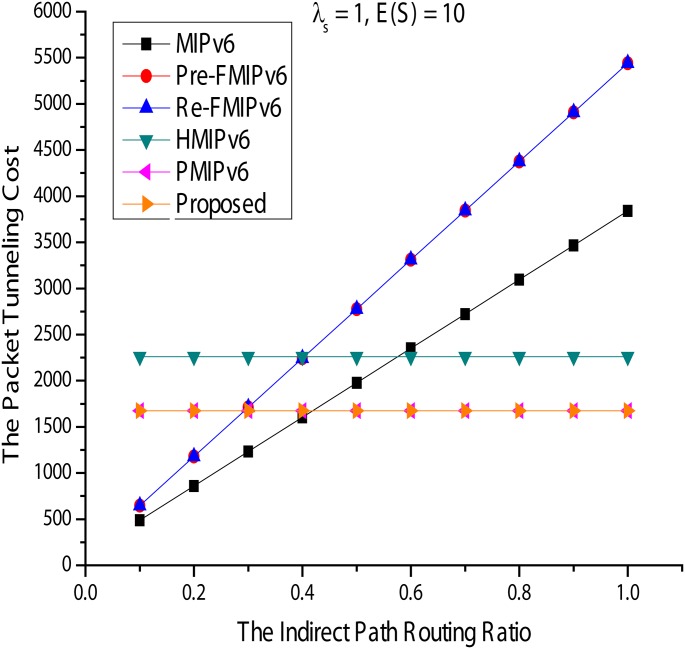
Packet Tunneling Cost for Indirect Path Routing Ratio.

Figs 18 and 19 show the costs of packet tunneling. In Fig 19, *ω* and E(S) are fixed at 0.2 and 10, respectively, compared with *λs* showing changes in packet tunneling costs. The result is similar to that of the packet transfer cost. Thus, even if applying a local mobility management structure for managing the movement of the MN in the PMIPv6 and the proposed method in the HMIPv6 domain, there is an increase in packet tunneling overhead. For example, data packets from the MN in HMIPv6 must go through to the MN in the MAP. RO in MIPv6 is impossible in FMIPv6; that is, when *ω* = 1, HMIPv6, PMIPv6, and the proposed method showed good performance.

#### Total costs

**Fig 20:**
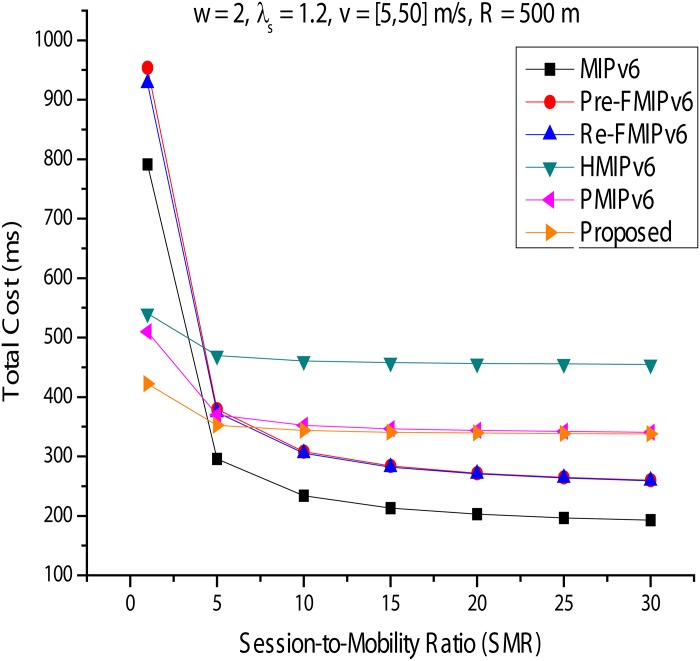
Total Cost for Session-to-Mobility Ratio (*ω* = 2, *λs* = 1.2).

**Fig 21:**
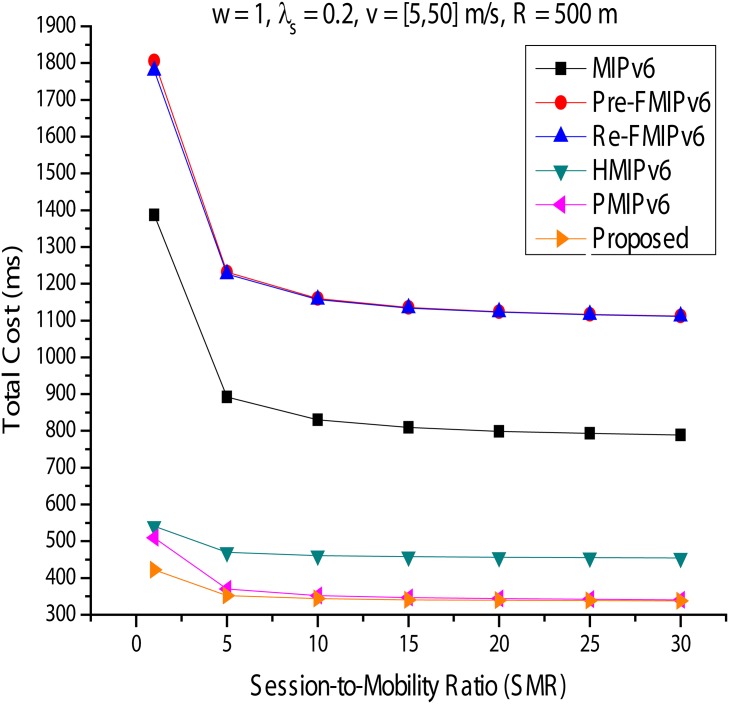
Total Cost for Session-to-Mobility Ratio (*ω* = 1, *λs* = 0.2).

In this section, we analyze the total cost for each mobility management protocol. *S*_*φ*_ is the mobility ratio, calculated by *λs*/*μc*(SMR), similar to the Call-To-Mobility ratio commonly used in performance evaluation studies of a wireless communication network. Fig 20 is a variation on the total cost of *ω* = 0.2, *λs* = 0.2, and *v* = [5, 50]*m*/*s* and is represented by *R* = 500*m*. With *S*_*φ*_ at a higher value, MIPv6, Predictive FMIPv6, and Reactive FMIPv6 all show good performance. This is because the high value of *S*_*φ*_ indicates a low mobility rate in the fixed session. This phenomenon becomes greater as *S*_*φ*_ is increased. HMIPv6, PMIPv6, and the proposed method reduce the cost of mobility signals in a high mobility rate environment. However, additional tunneling overhead is generated for the data packet delivery service for the MN. Next, *ω* is fixed at 1, and we again analyze the change in the total cost in Fig 21. HMIPv6 with PMIPv6 and the proposed technique can offer much better performance than other mobility management protocols in this analysis. As shown in Fig 21, this phenomenon was not affected by the increase in *S*_*φ*_. When the results shown in Figs 20 and 21 are assessed, HMIPv6, PMIPv6, and the proposed method have high mobility rates and one low-session work environment; we can see that they provide better performance than the other mobility management protocols. Additionally, with the method proposed in this paper, it can be seen that the higher the mobility ratio with the proposed method, the better the performance. However, if the RO is possible, MIPv6 and FMIPv6 show better performance in packet delivery owing to a significantly reduced cost.

## Conclusions

In this paper, we analyzed and compared existing IP mobility management protocols, including the virtual layer, based on PMIPv6 in terms of signal costs, packet transfer costs, packet tunneling costs, and total costs. In local mobility management, HMIPv6, PMIPv6, and the proposed scheme are regional approaches that manage the movement of the MN in a given mobility domain to reduce the number of mobility signaling messages for MN. That is, the movement of the MN is passed through the HA and CN. Thus, the signal costs will be significantly reduced in PMIPv6, the proposed scheme and HMIPv6 (Figs 14 and 15). RO should also be used to reduce the overhead of the data packet transmission. MIPv6, Predictive FMIPv6, and RO-enabled mobility management protocols, such as Reactive FMIPv6, showed better performance than HMIPv6, PMIPv6, and the proposed scheme. HMIPv6, PMIPv6 and the proposed scheme do not use an RO mechanism, and they use a tunneling technique for precisely maintaining the MN. However, additional tunneling overhead occurs at this time. The results can be seen in Figs 18 and 19. The performance of the proposed scheme can identify more clearly the improved performance of the other mobility scheme in this paper (Figs 20 and 21). Recently, mobile networks are increasing rapidly owing to performance improvements and rapid spread of mobile devices; quality improvement is required, including continuous service. Thus, this paper focused on mobility based on PMIPv6, one of the mobility management protocols. As shown in the performance analysis, according to the proposed scheme and a comparison, the proposed method provides better quality of service, minimizing the cost to the network, when handoff occurs during execution owing to the user’s movement, compared with other mobility management protocols. HMIPv6 and PMIPv6 both showed good performance when the performance was evaluated in different environments, and the ranking was graded according to a given condition. However, the proposed method always showed the best performance and the lowest total cost. The analysis performed in this study was used to identify the properties and performance evaluation elements of each mobility management protocol in addition to helping make decisions about consumer network design.

## Supporting Information

S1 File The Output Data of Performance Evaluation. (PDF)
