# Supplementary material for: Performance Analysis of Inter-Domain Handoff Scheme Based on Virtual Layer in PMIPv6 Networks for IP-Based Internet of Things
Source: PLoS One. 2017 Jan 27;12(1):e0170566. doi: 10.1371/journal.pone.0170566 (PMC5271355; doi:10.1371/journal.pone.0170566)
Supplement: S1 File — (PDF) [file pone.0170566.s001.pdf]

■ ***OUTPUT DATA***

Fig 14. Signal Cost for Velocity

Velocity	Pre-FMIPv6	Re-FMIPv6	MIPv6	PMIPv6	HMIPv6	Proposed
0	0.0	0.0	0.0	0.0	0.0	0.0
5	40.6	39.3	35.5	12.8	8.3	8.3
10	81.2	78.5	71.1	25.5	16.5	16.5
15	121.8	117.8	106.6	38.3	24.8	24.8
20	162.3	157.0	142.1	51.0	33.0	33.0
25	202.9	196.3	177.7	63.8	41.3	41.3
30	243.5	235.5	213.2	76.5	49.5	49.5

Fig 15. Signal Cost for Radius

Radius	Pre-FMIPv6	Re-FMIPv6	MIPv6	PMIPv6	HMIPv6	Proposed
400	201.6	194.3	172.7	41.4	15.3	15.3
450	178.2	171.9	153.4	35.7	12.5	12.5
500	159.3	153.5	137.0	31.3	10.1	10.1
550	143.8	138.3	122.8	27.2	8.2	8.2
600	131.0	125.9	112.0	24.1	6.7	6.7
650	120.1	115.4	102.3	21.3	5.3	5.3
700	110.7	106.4	94.2	19.1	4.1	4.1
750	102.7	98.6	87.2	17.1	3.2	3.2
800	95.7	91.7	81.1	15.4	2.3	2.3

Fig 16. Packet Delivery Dost for Session Arrival Rate

Session Arrival Rate	HMIPv6	PMIPv6	Proposed	Pre-FMIPv6	Re-FMIPv6	MIPv6
0	0.0	0.0	0.0	0.0	0.0	0.0
0.1	225.5	167.5	167.5	118.0	118.0	86.0
0.2	451.0	335.0	335.0	236.0	236.0	172.0
0.3	676.5	502.5	502.5	354.0	354.0	258.0
0.4	902.0	670.0	670.0	472.0	472.0	344.0
0.5	1127.5	837.5	837.5	590.0	590.0	430.0
0.6	1353.0	1005.0	1005.0	708.0	708.0	516.0
0.7	1578.5	1172.5	1172.5	826.0	826.0	602.0
0.8	1804.0	1340.0	1340.0	944.0	944.0	688.0
0.9	2029.5	1507.5	1507.5	1062.0	1062.0	774.0
1	2255.0	1675.0	1675.0	1180.0	1180.0	860.0

Fig 17. Packet Delivery Dost for Indirect Path Routing Ratio

Indirect Path Routing Ratio	Pre-FMIPv6	Re-FMIPv6	MIPv6	HMIPv6	PMIPv6	Proposed
0.1	544.0	544.0	384.0	2260.0	1670.0	1670.0
0.2	1088.0	1088.0	768.0	2260.0	1670.0	1670.0
0.3	1632.0	1632.0	1152.0	2260.0	1670.0	1670.0
0.4	2176.0	2176.0	1536.0	2260.0	1670.0	1670.0
0.5	2720.0	2720.0	1920.0	2260.0	1670.0	1670.0
0.6	3264.0	3264.0	2304.0	2260.0	1670.0	1670.0
0.7	3808.0	3808.0	2688.0	2260.0	1670.0	1670.0
0.8	4352.0	4352.0	3072.0	2260.0	1670.0	1670.0
0.9	4896.0	4896.0	3456.0	2260.0	1670.0	1670.0
1	5440.0	5440.0	3840.0	2260.0	1670.0	1670.0

Fig 18. Packet Tunneling Cost for Session Arrival Rate

Indirect Path Routing Ratio	Pre-FMIPv6	Re-FMIPv6	MIPv6	HMIPv6	PMIPv6	Proposed
0.1	544.0	544.0	384.0	2260.0	1670.0	1670.0
0.2	1088.0	1088.0	768.0	2260.0	1670.0	1670.0
0.3	1632.0	1632.0	1152.0	2260.0	1670.0	1670.0
0.4	2176.0	2176.0	1536.0	2260.0	1670.0	1670.0
0.5	2720.0	2720.0	1920.0	2260.0	1670.0	1670.0
0.6	3264.0	3264.0	2304.0	2260.0	1670.0	1670.0
0.7	3808.0	3808.0	2688.0	2260.0	1670.0	1670.0
0.8	4352.0	4352.0	3072.0	2260.0	1670.0	1670.0
0.9	4896.0	4896.0	3456.0	2260.0	1670.0	1670.0
1	5440.0	5440.0	3840.0	2260.0	1670.0	1670.0

Fig 19. Packet Tunneling Cost for Indirect Path Routing Ratio

Session Arrival Rate	HMIPv6	PMIPv6	Proposed	Pre-FMIPv6	Re-FMIPv6	MIPv6
0.1	226.0	167.5	167.5	118.0	118.0	86.0
0.2	452.0	335.0	335.0	236.0	236.0	172.0
0.3	678.0	502.5	502.5	354.0	354.0	258.0
0.4	904.0	670.0	670.0	472.0	472.0	344.0
0.5	1130.0	837.5	837.5	590.0	590.0	430.0
0.6	1356.0	1005.0	1005.0	708.0	708.0	516.0

0.7	1582.0	1172.5	1172.5	826.0	826.0	602.0
0.8	1808.0	1340.0	1340.0	944.0	944.0	688.0
0.9	2034.0	1507.5	1507.5	1062.0	1062.0	774.0
1	2260.0	1675.0	1675.0	1180.0	1180.0	860.0

Fig 20. Total Cost for Session-to-Mobility Ratio

Session-to-Mobility Ratio	Pre-FMIPv6	Re-FMIPv6	MIPv6	HMIPv6	PMIPv6	Proposed
0.0	954.0	928.0	791.0	541.0	509.0	422.0
5.0	380.0	374.0	297.0	470.0	370.0	353.0
10.0	309.0	306.0	235.0	461.0	352.0	344.0
15.0	285.0	282.0	213.0	457.0	346.0	340.0
20.0	272.0	270.0	202.0	456.0	343.0	339.0
25.0	266.0	263.0	195.0	455.0	342.0	338.0
30.0	261.0	259.0	193.0	454.0	341.0	337.0

Fig 21. Total Cost for Session-to-Mobility Ratio

Session-to-Mobility Ratio	Pre-FMIPv6	Re-FMIPv6	MIPv6	HMIPv6	PMIPv6	Proposed
0.0	1806.0	1780.0	1388.0	548.0	510.0	422.0
5.0	1234.0	1226.0	894.0	472.0	374.0	352.0
10.0	1162.0	1156.0	830.0	462.0	354.0	344.0
15.0	1136.0	1134.0	810.0	456.0	346.0	340.0
20.0	1116.0	1116.0	792.0	454.0	342.0	338.0
25.0	1112.0	1112.0	788.0	452.0	341.0	337.0
30.0	1111.0	1111.0	786.0	452.0	340.0	336.0
